# Two TGA Transcription Factor Members from Hyper-Susceptible Soybean Exhibiting Significant Basal Resistance to *Soybean mosaic virus*

**DOI:** 10.3390/ijms222111329

**Published:** 2021-10-20

**Authors:** Hua Jiang, Shengyu Gu, Kai Li, Junyi Gai

**Affiliations:** 1 Soybean Research Institute, Nanjing Agricultural University, Nanjing 210095, China; jianghua_5220@126.com (H.J.); gsynau@163.com (S.G.); kail@njau.edu.cn (K.L.); 2 MOA National Center for Soybean Improvement, Nanjing Agricultural University, Nanjing 210095, China; 3 MOA Key Laboratory of Biology and Genetic Improvement of Soybean (General), Nanjing Agricultural University, Nanjing 210095, China; 4 State Key Laboratory for Crop Genetics and Germplasm Enhancement, Nanjing Agricultural University, Nanjing 210095, China; 5 Jiangsu Collaborative Innovation Center for Modern Crop Production, Nanjing Agricultural University, Nanjing 210095, China

**Keywords:** soybean (*Glycine max* (L.) Merr.), TGA transcription factor, molecular characterization, *Soybean mosaic virus*, *Nicotiana benthamiana*, basal resistance

## Abstract

TGA transcription factors (TFs) exhibit basal resistance in *Arabidopsis*, but susceptibility to a pathogen attack in tomatoes; however, their roles in soybean (*Glycine max*) to *Soybean mosaic virus* (SMV) are unknown. In this study, 27 *TGA* genes were isolated from a SMV hyper-susceptible soybean *NN1138-2*, designated *GmTGA1*~*GmTGA27*, which were clustered into seven phylogenetic groups. The expression profiles of *GmTGAs* showed that the highly expressed genes were mainly in Groups I, II, and VII under non-induction conditions, while out of the 27 *GmTGAs*, 19 responded to SMV-induction. Interestingly, in further transient *N. benthamiana*-SMV pathosystem assay, all the 19 *GmTGAs* overexpressed did not promote SMV infection in inoculated leaves, but they exhibited basal resistance except one without function. Among the 18 functional ones, GmTGA8 and GmTGA19, with similar motif distribution, nuclear localization sequence and interaction proteins, showed a rapid response to SMV infection and performed better than the others in inhibiting SMV multiplication. This finding suggested that GmTGA TFs may support basal resistance to SMV even from a hyper-susceptible source. What the mechanism of the genes (*GmTGA8*, *GmTGA19*, etc.) with basal resistance to SMV is and what their potential for the future improvement of resistance to SMV in soybeans is, are to be explored.

## 1. Introduction

In general, plants show a resistance or susceptibility response in facing a pathogen attack, which usually involves dramatic changes of gene expression across multiple signaling and metabolism pathways [1,2]. Thereinto, a large number of genes that encode regulatory proteins were expropriated, among which transcription factors (TFs) are typical examples [3]. During plant growth and development, in defense responses and disease development, TFs often serve as transcriptional regulators in the nucleus and play a key role in manipulating gene expression by binding to specific *cis*-regulatory elements in the promoters of target genes [4].

TGA TF belong to Group D of the basic leucine zipper gene family, which is the largest TF family in plants [5]. This type of TF was first identified by their ability in binding to the activating sequence 1 element of the *Cauliflower mosaic virus* 35S promoter [6]. Because the activating sequence 1 was typically composed of a tandem of the sequence TGACG with approximately 21 bases in length, the TGACG-sequence-specific binding proteins were named as TGA proteins [7]. Initially, the sequence analysis of two TGA cDNA clones isolated from tobacco showed both proteins containing a basic leucine zipper (bZIP) domain composed of both basic and zipper regions [6]. The basic region usually contained the nuclear localization signal (NLS), which was rich in arginine and lysine, interacting with the major groove of the DNA double helix. While the zipper region was an amphipathic helix of 30–40 residues with every seventh residue a leucine, which was required to hold together two DNA binding regions [5]. The bZIP proteins commonly formed homodimers or heterodimers to perform their function, and specificity was determined by the non-leucine residues in the zipper region [5]. Since the first *TGA1a* gene was found in tobacco [6], many TGA TFs have been identified in plants, such as in the dicotyledonous plant *Arabidopsis*, there were ten members [8]. Similarly, four TGA TFs and 11 TGA-like proteins were also identified in the monocotyledonous plant rice [9].

After the advent of *Arabidopsis* as one of the prime model systems for plant molecular biology, the TGA family analysis was mainly performed in this species [10]. The TGA family was grouped into five clades according to the amino acid sequence similarity in *Arabidopsis* [5,10]. Among them, TGA1 and TGA4 belonged to clade I, TGA2, TGA5, and TGA6 belonged to clade II, TGA3 and TGA7 belonged to clade III, TGA9 and TGA10 belonged to clade IV, and PERIANTHIA/TGA8 belonged to clade V [10]. The clade II TGA TFs were identified as interaction partners of the ankyrin repeat protein NPR1 [NONEXPRESSOR OF pathogenesis-related (PR) GENES 1] to activate NPR1 target genes expression through directly binding to their *cis*-elements [11]. NPR1 was a key regulator of *PR* genes expression and was required to establish the systemic acquired resistance (SAR) [12]. The knockout analysis of *Arabidopsis TGA2*, *TGA5*, and *TGA6* revealed their redundant and positive roles in SAR, whereas the *TGA* triple knockout plants were not impaired in basal resistance to the bacterial pathogen *Pseudomonas syringaepv maculicola* ES4326 [12]. Though the clade I TGA TFs did not interact with NPR1 in the yeast two-hybrid system, they could interact in SA-treated leaves, and this suggested that TGA acted independently of NPR1 or on the upstream of SA biosynthesis and NPR1-dependent signaling to positively regulate basal resistance [13,14,15,16]. In contrast, TGA1.a-TF promoted disease development caused by *Botrytis cinerea* in tomato by suppressing the expression of two jasmonic acid-dependent defense genes, *proteinase inhibitors I* and *II* [17]. Furthermore, clade III TGA3 played a positive role in basal resistance, which recruited cytokinin-activated TF ARR2 to SA-responsive defense promoters through direct protein-protein interaction, while TGA7 does not [18,19]. In addition, clade IV TGA9 and TGA10 were redundantly required for anther development and were involved in ROS-mediated responses to bacterial PAMP flg22 [20,21]. Similarly, PERIANTHIA/TGA8 TF also played a role in flower development, which was a repressor of petal formation [22]. These findings indicated that TGA proteins played critical roles in pathogen-induced basal resistance, susceptibility and SAR (Figure S1), which suggested the homologous genes of *Arabidopsis* TGA TFs in different plant species may also be functional diversification.

Soybean (*Glycine max* (L.) Merr.) is an economically important oil and protein crop. *Soybean mosaic virus* (SMV) is a prevalent viral pathogen of soybean [*Glycine max* (L.) Merr.], found especially in China [23,24,25], usually leads to soybean yield reductions ranging from 8% to 100% [26]. SMV belongs to *Potyvirus*, its genome is a single-stranded, positive-sense (+) RNA, with a length of ~ 9.6 Kb. In general, SMV’s host range is restricted mostly to cultivated soybean and wild soybean [27]. Thereinto, the cultivated soybean *NN1138-2* is hyper-susceptible to 22 SMV strains identified in China [24]. Besides, *N. benthamiana* plants, as an ideal model plant for the investigation of plant–pathogen interaction was also systemically infected by some SMV isolates [28,29,30], which are helpful for studying the function of soybean endogenous genes to SMV stress.

To date, most TGA TFs studies have been carried out in model plant *Arabidopsis*, but there is a lack of reports on the potential functions (susceptibility, or resistance, or neutral) of soybean TGA TFs under SMV stress. In this study, we intend to isolate GmTGA TFs from an SMV hyper-susceptible soybean cultivar *NN1138-2*, classify them into different phylogenetic groups, detect their expression levels under non-inducing and SMV-inducing condition, and evaluate the function of *GmTGAs* to SMV challenge to understand the potential of GmTGA TFs for improving the self-defense of susceptible host soybean or promoting SMV to successfully colonize the host.

## 2. Results

### 2.1. Identification and Phylogenetic Groups of GmTGAs in Soybeans

We are interested in TGA TFs because the literature reported that these kinds of TFs were involved in the disease processes [16,17,19]. The characteristic analysis of gene families in soybean is often based on the sequences of the nucleic acid and amino acid derived from the reference genome of *Williams 82* [31]. Differently, we aim to uncover the characteristics of *GmTGA* gene family in the SMV-susceptible soybean cultivar *NN1138-2*. Firstly, BLASTP and HMM searches were performed against the reference protein of soybean *Williams 82* to identify *GmTGA*. As a result, 25 and 27 *GmTGA* genes were searched respectively. Twenty five out of 27 *GmTGA* genes were consistent and the remaining two genes were *GmTGA18* and *GmTGA21* (Table 1). Additionally, all identified TGA TFs contained classical bZIP and DOG1 domains based on SMART and Pfam analysis. Based on this, the coding sequences of 27 TGA TFs in cultivar *NN1138-2* were obtained by homology cloning, and the primers used were listed in Table S1. As shown in Table 1, all *GmTGAs* were named *GmTGA1 ~ 27,* depending on their location on the chromosomes and Gene ID number in soybean. These genes were widely distributed on 16 out of 20 chromosomes with a maximum of three *TGA* genes on chromosome 3, 10 and 13, respectively. Meanwhile, variations of the open reading frame, amino acid residues, molecular weight, and protein isoelectric points were predicted, it suggested the presence of putative novel variants in the gene family (Table 1).

**Figure 1:**
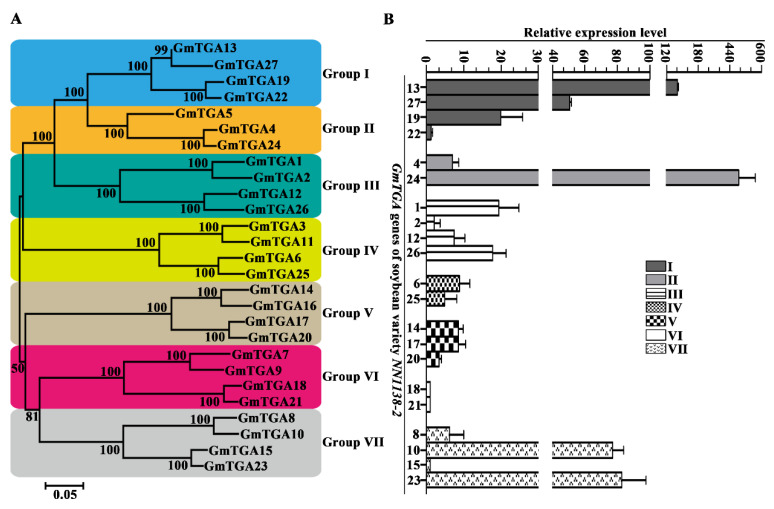
Phylogenetic relationship analysis of 27 GmTGA proteins of soybean *NN1138-2* and expression profile of these *GmTGA* genes in the unifoliate leaves. (**A**) Phylogenetic relationship analysis of 27 GmTGA proteins. The amino acid sequences of 27 GmTGA proteins from soybean *NN1138-2* were used to construct the neighbor-joining (NJ) tree using MEGA 6.0 with 1000 bootstrap replicates, from which 7 groups of GmTGA were identified. (**B**) Expression profile of *GmTGA* genes in fully developed unifoliate leaves tissue. The mRNA transcript levels of 27 *GmTGA* genes were analyzed using qRT-PCR in the unifoliate leaves. Out of them, the fluorescence signal of 21 *GmTGA* genes were detected while others not. *GmEF1B* and *GmActin11* were used as internal controls. All experiments were performed with three independent biological repeats. The error bars represent the standard deviation.

According to the phylogenetic tree of all 27 GmTGAs from *NN1138-2*, seven groups (I to VII) were clustered (Figure 1A), which was similar to the previous report on sub-classification within TGA family proteins [32]. GmTGA13, 19, 22 and 27 were assigned to Group I; GmTGA4, 5 and 24 were assigned to Group II; GmTGA1, 2, 12 and 26 were assigned to Group III; GmTGA3, 6, 11, and 25 were assigned to Group IV; GmTGA14, 16, 17 and 20 were assigned to Group V; GmTGA7, 9, 18 and 21 were assigned to Group VI; GmTGA8, 10, 15 and 23 were assigned to Group VII. Among them, only Group II contained three GmTGAs and others contained four GmTGAs. Besides, phylogenetic analyses showed a close relationship between soybean TGA members and *Arabidopsis* TGA family (Figure S2), while the addition of *Arabidopsis* TGA proteins did not affect the grouping of the phylogenetic tree (Figure S2). As expected, most of the *GmTGA* genes clustered in pairs, except *GmTGA5*, and Group I was the largest with seven members of soybean and *Arabidopsis* (Figure S2). In addition, a distinct phylogenetic branch, annotated as GmTGA4, 5 and 24, was not easily linked to any of the AtTGA orthologues, suggested that this group of TGA members were specific to the soybean genome.

### 2.2. Differential Expression Profiles of GmTGAs in the Unifoliate Leaves of Soybean

To explore expression patterns of seven groups of *GmTGA* genes in soybean plants at VC stage, qRT-PCR was performed using gene-specific primers. The relative expression level of 21 out of 27 *GmTGA* genes was shown in Figure 1B. Similar to the transcriptome data of soybean *Williams 82* in the phytozome database, the fluorescence signal of the *GmTGA3*, *5*, *7*, *9*, *11*, and *16* belonging to four different groups (II, IV, V and VI) were not detected. It indicated that these six genes were not expressed in the unifoliate leaves during the VC development stage of soybean. As shown in Figure 1B, there were differences in the relative expression level of seven groups of *GmTGA* genes. Among them, the genes in Groups I, II and VII were very highly expressed, but in Groups III, IV, V and VI they were not, especially in Group VI where they were almost not expressed. Moreover, the relative expression level also varied in the same group (Figure 1B). Additionally, out of 21 *GmTGA* genes, eight genes expressed relatively higher, that is, *GmTGA1*, *10*, *13*, *19*, *23*, *24*, *26* and *27*, five genes expressed relatively lower, that is, *GmTGA2*, *15*, *18*, *21* and *22*, and others were between them.

### 2.3. Differential Responses of GmTGA Genes to SMV Challenge

**Figure 2:**
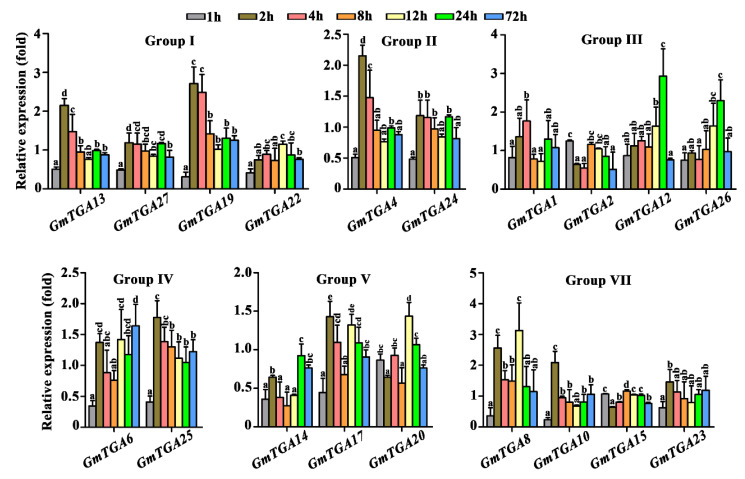
Expression profiles of *GmTGA* genes of soybean *NN1138-2* following infection with SMV at 1, 2, 4, 8, 12, 24 and 48 h post-inoculation (hpi). RNAs isolated from non-inoculated and inoculated unifoliate leaves of soybean were employed to analyze the expression of 7 groups of *GmTGA* genes using qRT-PCR. The relative expression levels were calculated by comparing the gene-expression values in inoculated vs. non-inoculated unifoliate leave tissues using the 2^−∆∆Ct^ method. The data are presented as mean ± standard deviation from three independent experiments. Relative quantitative values in the same gene with different letters are significantly different at *p* = 0.05 level.

In general, the unifoliate leaves were used for artificial SMV-inoculation in soybean. To investigate the potential roles of *GmTGA* genes in response to SMV challenge; the expression profiles of *GmTGA* genes in unifoliate leaves were examined at 1, 2, 4, 8, 12, 24 and 72 h post-inoculation (hpi) using qRT-PCR. The relative expression levels of 19 out of 21 *GmTGA* genes under Mock and SMV treatment were shown in Figure 2 and Figure S3. The cycle threshold value of both *GmTGA18* and *21* were consistent (equal to 35) at different time points before and after SMV inoculation, indicating Group VI was not involve in the SMV disease process. The transcript levels of *GmTGA* genes showed differences among and within the six TGA groups (I to V, and VII), which suggested the different transcriptional response of these *GmTGA* genes to SMV inoculation. At 1 hpi, most of the *GmTGA* genes were down-regulated and only *GmTGA2* and *GmTGA15* were slightly up-regulated. *GmTGA4*, *8*, *10*, *13* and *19* showed a rapid response to SMV inoculation at earlier 2 h time points; while *GmTGA12* and *26* showed an intense response at 24 hpi (Figure 2). In contrast, *GmTGA14* was down-regulated throughout each inoculation time point (Figure S3). Among them, *GmTGA13* and *19* belong to Group I, *GmTGA4* belong to Group II, *GmTGA12* and *26* belong to Group III, *GmTGA14* belong to Group V, and *GmTGA8* and *10* belong to Group VII, which indicated that Groups I, II, III, V and VII may be mostly involved in response to SMV-stress. Besides, *GmTGA1*, *6*, *17*, *20*, *22*, *24*, *25* and *27* were also induced. In short, our results suggest that a part of the *GmTGA* genes may be required for the host soybean self-defense or SMV successful colonization in soybean.

### 2.4. Transient Overexpression of GmTGA Genes in Nicotiana benthamiana Inhibiting but Not Promoting SMV-Multiplication

In a previous study, we found that *N. benthamiana* plants can be systemically infected by an SMV isolate 4278-1 [28]. The *N. benthamiana*-SMV-4278-1 pathosystem combined with *Agrobacterium*-mediated transient expression assay was a potential for exploring the function of *GmTGA* genes to SMV. Focusing on the putative importance of the *GmTGA* genes’ response to SMV-induction on improving or inhibiting SMV multiplication, the leaves of ~4 weeks *N. benthamiana* seedlings were infiltrated by *Agrobacterium tumefaciens* that contain each overexpression construct of the 19 *GmTGA* genes responded to SMV-induction, followed by the mechanical inoculation of the SMV isolate 4278-1, and the viral contents were further detected by ELISA assay at 3 dpi. The results showed 18 out of 19 *GmTGA* genes were positive for inhibiting SMV multiplication, except *GmTGA 6* with no effect on SMV multiplication compared to empty vector control (Table 2). Moreover, the effects of most overexpressed *GmTGA* genes were similar to positive control (SMV-*cp* gene overexpression) on inhibiting SMV multiplication for SMV-inoculated leaves in three independent experiments (Table 2). Among them, *GmTGA8* and *GmTGA19* had a superior performance to positive control and other *GmTGA* genes. The two genes, belonging to Groups I and VII, respectively, responded promptly to SMV infection and inhibited strongly to SMV multiplication (Figure 2 and Table 2). It is unexpected that no one promoted SMV multiplication in inoculated leaves, which indicated the *GmTGA* genes were not responsible for the susceptibility to SMV in hyper-susceptible soybean but benefiting for the basal resistance to SMV (Table 2). It also suggested that *GmTGA* genes may be functionally redundant for inhibiting SMV multiplication. Taken together, multiple *GmTGA* genes were recruited by the SMV-susceptible host soybean under SMV challenge and served a positive role in self-defense. Especially, the genes with rapid-response to SMV-infection, *GmTGA8 and 19*, may have certain potential for the improvement of SMV-resistance for SMV-susceptible soybeans.

### 2.5. Characteristics and Interaction Network of GmTGA 8 and GmTGA19 Proteins

**Figure 3:**
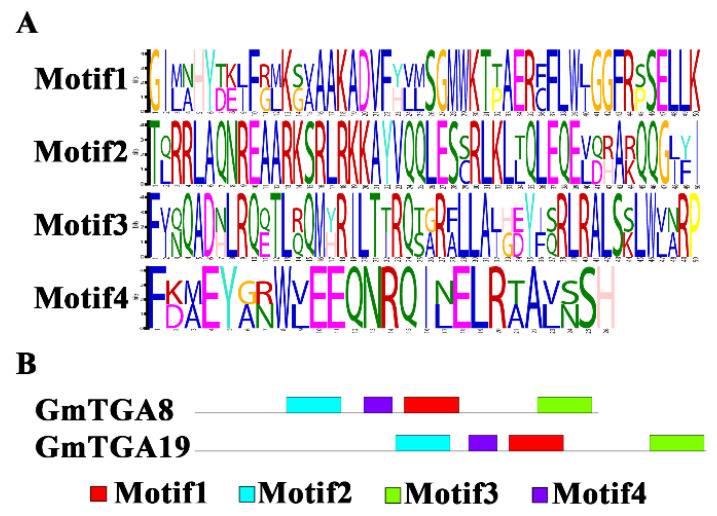
Conserved motifs in GmTGA 8 and 19 protein using MEME-suite. (**A**) The conserved motifs between GmTGA 8 and 19 proteins. The second motif covers the nuclear localization signals (NLS). Only the motifs with *E*-values < 0.05 as well as no overlap with each other were displayed. (**B**) The distribution of motifs along with the protein sequences. Gray lines represent the non-conserved sequences, and each motif is represented by a box numbered at the bottom.

In view of soybean genes, *GmTGA8* and *GmTGA19* were superior to others in inhibiting SMV-multiplication; their conserved motifs were predicted based on the MEME online tool, using “searching for ten motifs” and leaving the rest setting kept as default. The results showed that GmTGA8 and GmTGA19 contained four conserved motifs with a significance level of 0.05. As shown in Figure 3A, the width of motif 1 to motif 3 was 50, while for motif 4 it was 26. Among them, motif 2 showed the highest identity of 82%, and was rich in arginine (R) as well as glutamine (Q). Besides, we found that the two TGA proteins exhibited the same motifs in C-terminal regions (motifs 1 to 4), suggested the C-terminal part of TGA proteins was conserved, whereas the N-terminal region was divergent (Figure 3). Due to the TF acting in the nucleus, the NLS of GmTGA8 and GmTGA19 was predicted by the NLStradamus online tool [33], and the same nuclear localization sequence “REAARKSRLRKK” located in the motif 2 region was discovered among them.

**Figure 4:**
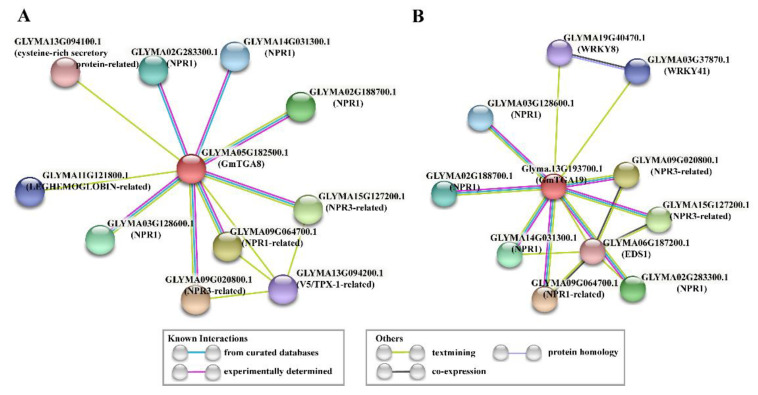
STRING interaction diagram of the analyzed GmTGA 8 (**A**) and 19 (**B**) in soybean. Each filled node denotes a gene and protein product of each gene is predicted based on phytozome online database; edges between nodes indicate interactions between protein products. Different edge colors represent the existence of different types of evidence used in predicting the associations. The first shell was set to no more than ten interactors and no interactor was set in second shell.

To identify the interaction proteins of GmTGA8 and GmTGA19 in soybean, the two TGA protein sequences as input were used for constructing the PPI networks through the STRING online tool (Figure 4). As shown in Figure 4A, the GmTGA8 protein showed interactions with 11 proteins, which were divided into six categories, including NPR1, NPR1-related, NPR3-related, cysteine-rich secretory protein-related, leghemoglobin-related and V5/TPX-1 related proteins. Similarly, the GmTGA19 protein showed interactions with ten proteins, which were divided into five categories, including NPR1, NPR1-related, NPR3-related, EDS1 and WRKY8/41 proteins (Figure 4B). As expected, the similar types of proteins were retrieved in both PPI networks, such as the NPR class proteins, in which NPR1 was a key regulator of the SA-mediated SAR in *Arabidopsis* [34]. In addition, we also found specific interaction proteins in both PPI networks, indicating that the regulatory network between GmTGA8 and GmTGA19 proteins were distinct. Thus, identifying the potential associated genes of *GmTGA8* and *GmTGA19* was helpful for understanding and exploring their possible regulatory mechanisms in soybean when suffering from SMV challenge.

## 3. Discussion

### 3.1. Characteristics of the GmTGA Family in Response to SMV Stress

In this study, a total of 27 *GmTGA* genes isolated from a SMV hyper-susceptible soybean *NN1138-2* were identified and analyzed for their characteristics and expression levels under non-inducing and SMV-inducing conditions. Obviously, the *TGA* gene number in soybean was more than that in other plant species, which might come from the expansion of the soybean gene family caused by soybean-specific segmental duplications [6,8,9,31]. In general, the large number of TGA genes could be interpreted as a possible functional redundancy and/or sub- or neo-functionalization of this protein family in soybean.

The expression patterns of *GmTGA* genes under different stress stimuli might provide insights into the functional divergence in soybean. The expression of the *GmTGA* genes in soybean *NN1138-2* varied at the soybean VC development stage, which indicated that some GmTGA proteins may be recruited for tissue development (Figure 1). *Arabidopsis* TGA proteins often played a vital role in growth development, such as *AtTGA9* and *AtTGA10,* which were expressed throughout early anther primordia and redundantly required for another development [20]. However, unlike *Arabidopsis*, there have been barely any reports regarding *GmTGA* genes regulating plant development in soybean.

Additionally, TGA TFs responded differentially to SMV-infection, some *GmTGA* genes *(GmTGA4*, *8*, *10*, *13*, and *19*) showed a rapid response, while some (*GmTGA12* and *26*) were slower (Figure 2 and Figure S3). This meant that their responding mechanisms were diverse, and were similar to the mechanisms of stress response of rice bZIP family genes [35]. More importantly, the response of the TGA members of Groups I, II, III, V and VII to SMV infection in the unifoliate leaves provided evidence of their novel function as a regulator of the virus stress process (Figure 2 and Figure S3). Besides, due to *AtTGA1*, *2*, *4*, *5* and *6* were involved in the plant defense response in *Arabidopsis* [5], the closer the phylogenetic relationships between soybean TGA TFs were in Groups I and VII (Figure 1 and Figure S2), and they might also be required for self-defense in soybean or SMV successful colonization in a host. These findings provided a reference for the response of *GmTGA* genes in a virus-susceptible soybean in the face of infection by a single-stranded RNA virus.

### 3.2. Potentials of GmTGA8 and GmTGA19 in Improving Soybean Resistance to SMV

In the present study, against our expectations, most *GmTGA* genes from a hyper-susceptible soybean inhibited SMV multiplication in inoculated leaves of *N. benthamiana* (Table 2). It indicated that *GmTGA* genes were not responsible for the susceptibility of hyper-susceptible soybean to SMV, but for the basal resistance to SMV, and they seemed to be functionally redundant for coping with SMV stress. In *Arabidopsis*, the knockout of *Arabidopsis TGA2*, *TGA5* and *TGA6*, which belonged to the same group, revealed their redundant and essential roles in SAR [12]. While in soybean, the functional GmTGA proteins belonged to different groups in a phylogenetic relationship (Figure S2), suggesting the functional differentiation of the *TGA* paralogous genes in soybean was not obvious in response to an SMV attack. In contrast, tobacco bZIP TF TGA2.2 and TGA2.1 had distinct roles in the plant defense response and plant development, which indicated that tobacco TGA TFs possessed functional diversity [36]. In addition, overexpression of AtTGA2 conferred partial resistance to soybean cyst nematode in transgenic soybean hairy roots, AtTGA9 and AtTGA10 were involved in ROS-mediated responses to bacterial PAMP flg22 [21,37]. However, the reports on the functions of GmTGA TFs under pathogen attack or abiotic stress were very limited; for instance, *GmTGA17* enhanced tolerance to drought and salt stress in both transgenic *Arabidopsis* plants and soybean hairy roots [38]. Thus, the function of *GmTGA* paralogous genes for growth development or resistance/tolerance to different stresses need to be further studied. In fact, we found that the *GmTGA* genes exhibited various degrees in inhibiting SMV replication (Table 2). Compared to the overexpression of the SMV coat protein, which increased the resistance to SMV in transgenic soybean [39], GmTGA8 and GmTGA19 performed better on inhibiting SMV multiplication than others. Therefore, TGA TFs possessed special roles in pathogen attack, and it was worthwhile to further study the function of GmTGA8 and GmTGA19 by transgene in soybean.

It was not difficult to find that GmTGA 8 was grouped with AtTGA1 and AtTGA4, and GmTGA19 was grouped with AtTGA2, AtTGA5 and AtTGA6 according to the phylogenetic relationship (Figure S2). As known to all, the *Arabidopsis* TGA TFs (TGA2, TGA5 and TGA6) play positive roles in the self-defense response, which interacted with NPR1 (NONEXPRESSOR OF PR GENES 1) to regulate the expression of *PR-1* for gaining SAR [11,12]. Similarly, AtTGA1 and AtTGA4 also participated in plant immunity independent of NPR1 mediated SA-dependent defense response [13,14,15]. Although it was not clear whether the resistance mechanism of GmTGA8 and GmTGA19 in soybean was similar to that in *Arabidopsis* (Figure S1), the results of the predicted protein interaction network indirectly supported that NPR1 was the potential partner of GmTGA8 and GmTGA19 (Figure 4), and implied that *GmTGA* genes may also rely on the NPR1 pathway. Notably, WRKY8 and WRKY41 were the candidate partners of GmTGA19 rather than GmTGA8, and this WRKY-TGA pair was also involved in activating the expression of SA-dependent defense genes in the charge of SAR in *Arabidopsis* [40,41]. The mechanism of *GmTGA* genes regulating the SMV disease process needs to be explored in the next step. Additionally, what the functions of *GmTGA* genes are in hyper-resistant soybean needs to be explored in the future.

## 4. Materials and Methods

### 4.1. Plant Materials and SMV Strain

SMV-susceptible soybean cultivar *NN1138-2* was grown in an insect-free greenhouse with a 16 h photoperiod at 25 °C. SMV isolate 4278-1 was provided by the National Center for Soybean Improvement (Nanjing Agricultural University, Nanjing, China).

### 4.2. Identification, Cloning and Sequence Analysis of GmTGA

The amino acid sequences of *Arabidopsis* TGA TFs were retrieved from the *Arabidopsis* Information Resource (https://www.Arabidopsis.org, accessed on 17 June 2018). Two different approaches were used to mine the TGA TFs in soybean: (i) the BLASTP search (e-value 1 × 10^−20^) was performed using *Arabidopsis* TGA TFs sequences; (ii) the Hidden Markov model (HMM) scan was performed using an HMM profile, which was built by the alignment of a set of *Arabidopsis* TGA TFs sequences. A final unique set of the protein sequences identified using the above approaches was further scanned for the presence of bZIP and DOG1 domains based on SMART [42] and Pfam [43] analysis. Only the sequences containing these domains were retained. For cloning the TGA TFs from soybean cultivar *NN1138-2*, the total RNA of the leaf, stem, root hairs and seed were separately extracted using the RNA isolation kit (Tiangen, Beijing, China) and the RNA mixture served as the template for reverse transcription using the PrimeScript™ II 1st strand cDNA synthesis kit (Takara, Dalian, China). The PCR reaction was performed using Phanta Max Super-Fidelity DNA polymerase (Vazyme, Nanjing, China) according to the recommended procedure. Another alternative amplification method was adopted while the PCR products could not be obtained from cDNA, that is, the DNA instead of the cDNA served as the template of the PCR reaction. The DNA was extracted using the DNAsecure plant kit (Tiangen, Beijing, China). The above primers used were listed in Table S1. All PCR products were purified using a gel extraction kit (Omega, Guangzhou, China) and were further ligated to the T vector using the pClone007 blunt vector kit (Tsingke, Nanjing, China) for sequencing (General Biosystems, Chuzhou, China). The coding sequences of all TGA TFs derived from *NN1138-2* were predicted through comparing to the corresponding sequences of TGA TFs in *Williams 82*. The molecular weights and theoretical isoelectric points of TGA proteins were predicted by the ExPASy server [44].

### 4.3. Phylogenetic Analysis

Multiple alignments of the GmTGA amino acid sequences were performed using ClustalW with the default options in MEGA Version 6.0 [45,46]. Phylogenetic trees were constructed based on the neighbor-joining (NJ) method with the p-distance model and maximum likelihood analysis with a bootstrap of 1000 replicates.

### 4.4. SMV Inoculation and Sample Collection

SMV isolate 4278-1 was increased and maintained on a highly susceptible soybean *NN1138-2*. Young symptomatic leaves were homogenized in 0.01 M phosphate buffer (PH 7.4) using a sterilized mortar with pestle and the tissue homogenates were filtered with two layers of cheesecloth. The fully developed unifoliate leaves of the soybean cultivar *NN1138-2* were further mechanically inoculated by the above-mentioned filtered sap mixed with a small amount of carborundum powder (600-mesh) and the mock (0.01 M phosphate buffer, PH 7.4) inoculation as control. The inoculated leaves were collected at 0, 1, 2, 4, 8, 12, 24 and 72 h post-inoculation (hpi), respectively. Meanwhile, the fully developed unifoliate leaves of some untreated soybean plants were also collected. All the samples were stored at −80 °C immediately after freezing in liquid nitrogen.

### 4.5. The Quantitative Real-Time PCR Analysis

The total RNA was extracted from the untreated and treated unifoliate leaves of soybean cultivar *NN1138-2* using an RNA isolation kit (Tiangen, Beijing, China). The corresponding first-strand cDNA was synthesized (PrimeScript^TM^ RT reagent Kit, TAKARA, Dalian, China) and used for the quantitative real-time polymerase chain reaction (qRT-PCR). The qRT-PCR was performed using a LightCycler 480II(ROCHE) in conjunction with the SYBR^®^ *Premix Ex Taq*™ II (Tli RNaseH Plus, TAKARA, Dalian, China). Each reaction consisted of 5 μL of SYBR^®^ *Premix Ex Taq*™ II, 0.2 μL of each 10 μM gene-specific forward primer and reverse primer, 2 μL cDNA in a final volume of 10 μL. The primer pairs used for qRT-PCR are shown in Table S2 and the primers of the reference genes *GmEF1B* and *GmActin 11* are taken from the literature [47]. The protocol was as follows: 30 s of initial denaturation at 95 °C; the samples were then subjected to cycling parameters of 95 °C for 5 s (heating rate: 4.4 °C/s), 60 °C for 30 s (heating rate: 2.2 °C/s) (for 40 cycles). The relative expression of the gene was calculated using the 2^−∆∆Ct^ method [48]. Three technical replicates in each of the three biological replicates for each sample were taken for qRT-PCR analysis.

### 4.6. Construction of Transient Overexpression Constructs

The vector pEarleygate103 was digested with restriction enzymes XhoI and PmlI, then the green fluorescent protein (*GFP*) gene and SMV coat protein (*cp*) gene were cloned into the pEarleygate103 vector by the one-step recombination method, respectively (ClonExpress II One Step Cloning Kit, Vazyme, China), that is, p103-GFP (empty vector) and p103-CP (positive control). Moreover, the coding sequences of individual *GmTGA* genes were amplified from the above-mentioned T vectors (pClone007 blunt vector), which contain all *GmTGA* genes sequences using their corresponding primers (Table S3), and were further cloned into the p103-GFP vector using the same restriction enzyme cutting site by the one-step recombination method, that is, the p103-*GmTGA genes* constructs.

### 4.7. Transient Assay in N. benthamiana, SMV Inoculation and ELISA Detection

For *Agrobacterium*-mediated transient overexpression of candidate genes in *N. benthamiana*, an individual overexpression construct was transformed into the *Agrobacterium tumefaciens* strain GV3101 through electroporation and the bacterial cultures were grown overnight (28 °C, 200 rpm), pelleted by centrifugation and then resuspended in 10 mM MgCl_2_. The suspension was diluted to OD600 = 0.5 with a solution containing a final concentration of 10 mM MgCl_2_, 10 mM MES pH 5.7, and 150 μM acetosyringone and was then infiltrated into 4-week-old leaves of *N. benthamiana* with a 1 mL needleless syringe. For SMV inoculation, the SMV isolate 4278-1-infected soybean leaves were homogenized in 0.01 M phosphate buffer (PH 7.4) in a grinder and the tissue homogenates were filtered with two layers of cheesecloth. Then, *Agrobacterium*-infiltrated leaves of *N. benthamiana* were mechanically inoculated with the filtered sap. For ELISA assay, the SMV antiserum (polyclonal rabbit antibodies) of ELISA was purchased from ACD Inc. (cat #V094-R1, Beijing, China) and the manufacturer’s instructions were followed for the next steps.

### 4.8. Conserved Motifs and NLS Analysis in GmTGA Protein

Multiple expectation maximization for motif elicitation (MEME) was employed to identify and analyze the conserved motifs of the GmTGA sequences and only the motifs with E-values < 0.05 as well as with no overlap with each other were reported. The NLS was predicted by the NLStradamus online tool [33].

### 4.9. Protein-Protein Interaction (PPI) Network Analysis

The search tool for the retrieval of the interacting genes (STRING) (https://string-db.org, accessed on 1 March 2021) database was applied to predict functional interactions of proteins [49]. Active interaction sources, including databases, experiments, neighborhood, gene fusion, co-occurrence, textmining and co-expression as well as species limited to “*Glycine max*” and an interaction score > 0.4 were applied to construct the PPI networks.

### 4.10. Statistical Analysis

The ELISA and qRT-PCR data were analyzed for their means, standard deviations, and single-factor analyses of variance using the SPSS software (version 18.0). Duncan’s new multiple range tests were performed to determine any significant difference among various treatments using the significance level of α = 0.05.

## 5. Conclusions

A total of 27 GmTGA genes isolated from an SMV hyper-susceptible soybean *NN1138-2* were identified through homologous cloning. Expression analysis showed that most of them participated in response to SMV stress. Transient overexpression of SMV responding to GmTGA genes in N. benthamiana exhibited the basal resistance to SMV rather than promoting SMV-multiplication. It indicated that GmTGA genes were not responsible for the susceptibility of hyper-susceptible soybean to SMV, but for the basal resistance to SMV. For GmTGA8 and GmTGA19 especially, they possessed similar motifs distribution and a nuclear localization sequence. Moreover, according to protein–protein networks prediction analysis, NPR-class proteins were their main partners. Taken together, the unveiling of the roles of soybean TGA genes for inhibiting SMV multiplication provided new insights into improving SMV-resistance in susceptible soybeans.

## Figures and Tables

**Table 1:** Characteristics and nomenclature of soybean TGA transcription factors in soybean cultivar *NN1138-2*.

Gene Name	Gene Model Name (Wm82.a2.v1)	Location Coordinates	ORF (bp)	Size (aa)	MW (kDa)	p*I*
*GmTGA1*	Glyma.01G084200	Chr01:24682405..24689210 (−)	1461	486	54.31	7.32
*GmTGA2*	Glyma.02G097900	Chr02:8834516..8841154 (−)	1404	467	51.85	7.32
*GmTGA3*	Glyma.02G176800	Chr02:29019977..29028838 (−)	1455	484	53.93	7.77
*GmTGA4*	Glyma.03G127600	Chr03:34171878..34178336 (−)	1383	460	50.93	7.00
*GmTGA5*	Glyma.03G128200	Chr03:34259590..34264026 (+)	873	290	32.37	8.82
*GmTGA6*	Glyma.03G142400	Chr03:35810204..35817330 (−)	1476	491	55.20	8.22
*GmTGA7*	Glyma.04G254800	Chr04:52132354..52137159 (−)	1089	362	40.93	8.69
*GmTGA8*	Glyma.05G182500	Chr05:37029299..37037313 (+)	1113	370	42.08	7.11
*GmTGA9*	Glyma.06G107300	Chr06:8630095..8635111 (+)	1068	355	39.98	6.21
*GmTGA10*	Glyma.08G140100	Chr08:10719899..10725773 (+)	1140	379	43.11	7.78
*GmTGA11*	Glyma.10G092100	Chr10:12738719..12747206 (+)	1554	517	57.92	6.61
*GmTGA12*	Glyma.10G276100	Chr10:49858890..49863059 (−)	1371	456	50.99	6.08
*GmTGA13*	Glyma.10G296200	Chr10:51306677..51314923 (−)	999	332	37.14	8.94
*GmTGA14*	Glyma.11G183700	Chr11:25149986..25158384 (+)	1476	491	54.01	6.04
*GmTGA15*	Glyma.11G236300	Chr11:33112560..33118319 (−)	1095	364	41.18	6.28
*GmTGA16*	Glyma.12G088700	Chr12:7269137..7277440 (−)	1506	501	55.25	6.61
*GmTGA17*	Glyma.12G184500	Chr12:34579255..34588903 (−)	1467	488	54.12	6.75
*GmTGA18*	Glyma.13G085100	Chr13:19659235..19664662 (−)	1113	370	41.92	7.13
*GmTGA19*	Glyma.13G193700	Chr13:30682181..30695761 (−)	1410	469	51.99	5.98
*GmTGA20*	Glyma.13G316900	Chr13:41139769..41147330 (+)	1473	490	54.63	6.96
*GmTGA21*	Glyma.14G167000	Chr14:41307404..41312938 (−)	1113	370	41.87	8.26
*GmTGA22*	Glyma.15G232000	Chr15:43630502..43639894 (−)	1344	447	49.45	5.86
*GmTGA23*	Glyma.18G020900	Chr18:1529888..1535807 (+)	1089	362	41.05	7.78
*GmTGA24*	Glyma.19G130200	Chr19:38989153..38995505 (−)	1380	459	50.72	8.53
*GmTGA25*	Glyma.19G145300	Chr19:40604030..40611501 (−)	1473	490	55.20	8.51
*GmTGA26*	Glyma.20G113600	Chr20:35557820..35562522 (+)	1368	455	50.70	5.88
*GmTGA27*	Glyma.20G246400	Chr20:47647701..47654066 (−)	1335	444	49.51	5.91

Note: ORF, open reading frame; MW, molecular weight; p*I*: isoelectric point.

**Table 2:** The virus content analysis in *N. benthamiana* leaves overexpressed with different *GmTGA* genes under SMV inoculation by DAS-ELISA at 3dpi.

	Sample Name	EV	SMV-Induced GmTGA Transcription Factors																			+
			Group I				Group II		Group III				Group IV		Group V			Group VII				
Treatment			#13	#27	#19	#22	#4	#24	#1	#2	#12	#26	#6	#25	#14	#17	#20	#8	#10	#15	#23	
Mock		0.10	0.11	0.13	0.12	0.11	0.10	0.12	0.12	0.12	0.12	0.12	0.11	0.12	0.11	0.12	0.12	0.12	0.12	0.11	0.13	0.12
SMV	E1	0.74 ^d^	0.41 ^bc^	0.40 ^bc^	0.28 ^a^	0.43 ^bc^	041 ^bc^	0.45 ^c^	0.47 ^c^	0.41 ^bc^	0.47 ^c^	0.45 ^c^	0.74 ^d^	0.45 ^c^	0.41 ^bc^	0.47 ^c^	0.46 ^c^	0.30 ^a^	0.42 ^bc^	034 ^ab^	0.51 ^c^	0.40 ^bc^
	E2	0.81 ^c^	0.49 ^b^	0.48 ^b^	0.32 ^a^	0.51 ^b^	0.49 ^b^	0.52 ^b^	0.55 ^b^	0.48 ^b^	0.55 ^b^	0.53 ^b^	0.80 ^c^	0.53 ^b^	0.48 ^b^	0.54 ^b^	0.53 ^b^	0.32 ^a^	0.50 ^b^	0.46 ^b^	0.58 ^b^	0.47 ^b^
	E3	0.72 ^c^	0.42 ^b^	0.45 ^b^	0.28 ^a^	0.41 ^b^	0.43 ^b^	0.40 ^b^	0.40 ^b^	0.39 ^b^	0.40 ^b^	0.46 ^b^	0.70 ^c^	0.44 ^b^	0.44 ^b^	0.45 ^b^	0.46 ^b^	0.27 ^a^	0.43 ^b^	0.40 ^b^	0.51 ^b^	0.42 ^b^

The different letters a, b, c and d represent the significance of the ELISA means at *p* = 0.05 level among treatments in a same row; Mock: 0.01 M phosphate buffer inoculation as control; SMV: isolate 4278-1; E1, E2 and E3: biological repeat 1, 2 and 3; EV: empty vector; #13, 27, 19, 22, 4, 24, 1, 2, 12, 26, 6, 25, 14, 17, 20, 8, 10, 15, and 23: overexpression construct of different *GmTGA* genes; +: overexpression construct of SMV coat protein gene; dpi: days post-inoculation.

## Data Availability

All data are available upon reasonable request.

## Supplementary Materials

The following are available online at https://www.mdpi.com/article/10.3390/ijms222111329/s1, Figure S1: Schematic representation of TGA TFs involved in pathogen-induced basal resistance, susceptibility and systemic acquired resistance (SAR). Figure S2: Phylogenetic analyses of TGA proteins from Arabidopsis and soybean. Figure S3: Expression profiles of GmTGA genes of soybean NN1138-2 before and after SMV inoculation at 1, 2, 4, 8, 12, 24 and 72 h post-inoculation (hpi). Table S1: The primers are used to amplify TGA TFs from soybean cultivar NN1138-2. Table S2: The qRT-PCR primers for the 27 GmTGA genes of soybean cultivar NN1138-2. Table S3: The construction primers of transient overexpression constructs.

## Abbreviations

$$\begin{array}{ll} \mathrm{TF} & \mathrm{transcription factor}\\ \mathrm{SMV} & \mathrm{Soybean mosaic virus}\\ \mathrm{N. benthamiana} & \mathrm{Nicotiana benthamiana} \end{array}$$
