# Supplementary material for: Two TGA Transcription Factor Members from Hyper-Susceptible Soybean Exhibiting Significant Basal Resistance to Soybean mosaic virus
Source: Int J Mol Sci. 2021 Oct 20;22(21):11329. doi: 10.3390/ijms222111329 (PMC8583413; doi:10.3390/ijms222111329)
Supplement: Supplementary file 1 [file ijms-22-11329-s001.zip › ijms-1411004-supplementary.pdf]

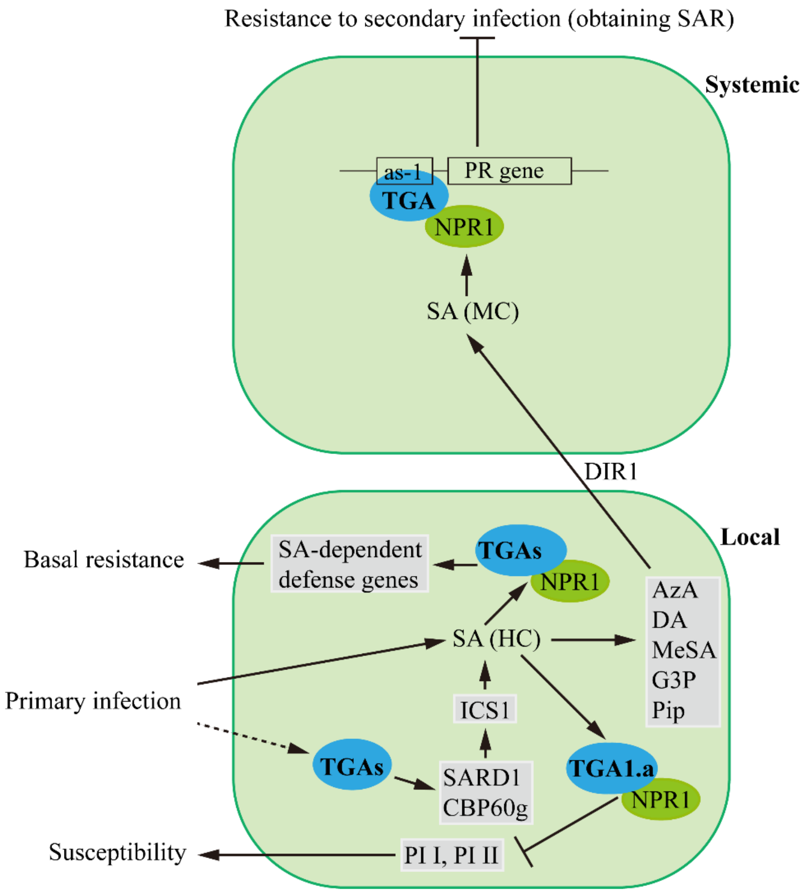

Figure S1. Schematic representation of TGA TFs involved in pathogen-induced basal resistance, susceptibility and systemic acquired resistance (SAR). A primary local infection may trigger the production of the immune signal salicylic acid (SA) or TGA TFs, and TGA TFs are located in upstream or downstream of SA. Upon pathogen infection, TGA TFs are activated and contribute to the induction of systemic acquired resistance deficient 1 (SARD1) and Calmodulin-binding protein 60g (CBP60g). SARD1 and CBP60g subsequently activate the expression of isochorismate synthase 1 (ICS1) which leads to increased SA biosynthesis. Moreover, SA also be directly elevated under pathogen infection. After that, NPR1 interact with TGA TFs to activate the expression of SA-dependent defense genes or suppress the expression of *proteinase inhibitors I* and *II* (*PI I* and *PI II*), which show the basal resistance or susceptibility to primary infection in local tissue. The production of mobile immune signals involved in azelaic acid (AzA), abietane diterpenoid dehydroabietinal (DA), methyl salicylic acid (MeSA), glycerol-3-phosphate (G3P), and pipecolic acid (Pip), which require a putative lipid-transfer protein (DIR1) for their functions. In distal organs, the intermediate level of SA lead to the accumulation of NONEXPRESSOR OF pathogenesis-related (PR) GENES 1 (NPR1). NPR1 can interact with TGA TFs to activate the expression of pathogenesis-related protein such as PR1, PR2, and PR5, which show the resistance to secondary infection in systemic tissue, i. e. obtaining SAR. SA (HC): high concentration of salicylic acid, SA (MC): medium concentration of salicylic acid, as-1:

activating sequence 1, solid line arrows represent direct activation regulation, dashed line arrows represent indirect activation regulation and blocked arrows indicate repression.

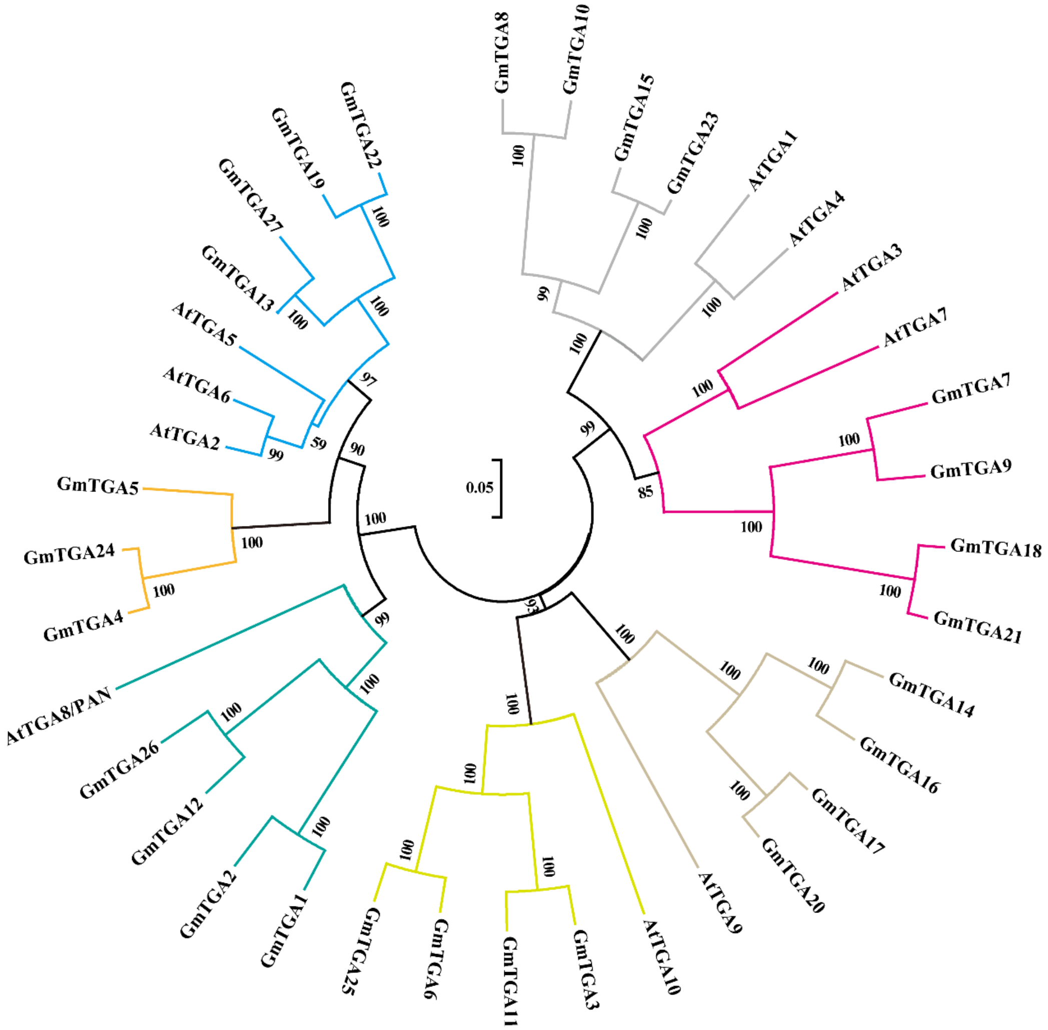

Figure S2. Phylogenetic analyses of TGA proteins from *Arabidopsis* and soybean. Each group is marked by a separate color.

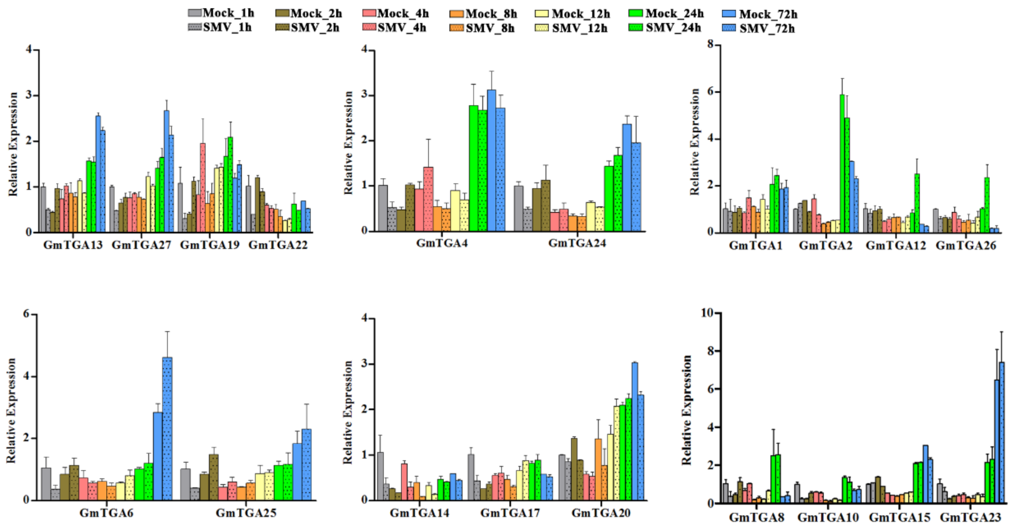

Figure S3. Expression profiles of *GmTGA* genes of soybean *NN1138-2* before and after SMV inoculation at 1, 2, 4, 8, 12, 24 and 72 h post-inoculation (hpi). RNAs isolated from non-inoculated and inoculated unifoliate leaves of soybean were employed to analyze the expression of 7 groups of *GmTGA* genes using qRT-PCR. The relative expression levels were calculated using the $2^{-\Delta\Delta Ct}$ method. Mock-inoculated soybean leaves at 1 h as the control (relative expression = 1). Data are presented as mean ± standard deviation from three independent experiments.

Table S1. The primers are used to amplify TGA TFs from soybean cultivar *NN1138-2*.

Name	Primer sequence (5'to 3')	Size(bp)	Template
GmTGA1	CAGTGTGTGACTGTGTCTGA/GAGCCAACATTGGAAGTCCT	1643	cDNA
GmTGA2	CTGCTGCTGCTTTTGTAACG/TGCAATGCTACCTGATTGTTG	1827	cDNA
GmTGA3	AGAAGGCGGTTTCACCATTA/ATGGGAAAGTCCAGAGGGAG	1752	cDNA
GmTGA4	GGGTTTGCAGGGCCATTATT/AACACACACCACGAAACAAGT	2053	cDNA
GmTGA5	ATGGGTAGTGCAGAAACTTTA/TCACTCCCTTGGCCTG	873	cDNA
GmTGA6	GTTTCGCCATTGATGAGAGC/TGATATGCAGGGGGAGAGAG	1691	cDNA
GmTGA7	CCTATAGAAAACTCTTTTATT/ACACGGCATTAACTCATTCA	1238	cDNA
GmTGA8	GTAGCAGCGGAGGATTTCTG/AGCCGGAACAGGATGATAAG	1271	cDNA
GmTGA9	ATTTGGGCGTTGCAAGAGAT/TTCCACTACATGTCTCTCCTT	1520	cDNA
GmTGA10	ATGTGTATGCACCGAATCGC/GCAATAGACATATCCTTTCAGG	1445	cDNA
GmTGA11	TCTATATCTCTTTGCGTGGAC/TCGTTATTATCATCACCACCAC	1653	DNA
GmTGA12	GCAACACCACACTACAGTCC/GGCACAATATCCGCTTGACA	1680	cDNA
GmTGA13	TCAAATCTGGTCAGTGGTGATA/TCATCTAAATTCCTTCAACTGTGT	1276	cDNA
GmTGA14	TATGTATTACTTTGACAGGA/ACATAAGAGACACGAGAGGA	1763	cDNA
GmTGA15	TAGGAGTATGTGTTGAATGA/ATATACACCATGCGTCCAAA	1378	cDNA
GmTGA16	TGAGAATGGTCTAAGAAGCC/CTGACACTCTTTCCATTCCT	1618	cDNA
GmTGA17	TCCCTAAAACACACATAGAGCTT/GAGCTTCCTCATCATGCACA	1736	cDNA
GmTGA18	GTTTGTATTGGCCAGATAGGGT/TTGTGCAAACCACATTACAGT	1249	cDNA
GmTGA19	GAATTGTTGATGTGGTGGTGAG/GGAAAACTATGGCAACTGAAACA	1735	cDNA
GmTGA20	TAGAGGTAAGCTTCAGGGAA/CTTCTTTCAGGCTAATGGGT	1261	cDNA
GmTGA21	TCTGAGGTTGAGGTTTCATTC/GTAGATTTGAGCACCTGTTGTG	1343	DNA
GmTGA22	GACAAGGGAGCTGAAAGAGG/ ACTGACTAGCATTTTGAACCCA	1936	cDNA
GmTGA23	TCCTGCCACCCTTCTATTCT/TGCGCTCTTGTAACCTTGTT	1303	cDNA
GmTGA24	ATTTGGGTTGCTCCCCTTTT/AGTCTTTAAGGGGGTCAGGT	1554	cDNA
GmTGA25	AGAAGACACAGAAGGCGCTA/TGCAGAAAAGAACCAGGAAGG	1807	cDNA
GmTGA26	ACACCTCATCCTCACTACCAT/ACGCATAACATTGGCACAAC	1587	cDNA
GmTGA27	AAAAGGGAACAGCAAGAGGG/CCCTTCAACTGTGTTACAATGG	1578	cDNA

Table S2. The qRT-PCR primers for the 27 *GmTGA* genes of soybean cultivar *NN1138-2*.

**Name**	**Primer sequence (5'to 3')**	**Size(bp)**
GmTGA1	GGCTTGGAAGCATTGCAACA/AGTGCCATTTGGCCCATGTA	104
GmTGA2	GGCATGGAAGCATTGCAACA/GAGGTCGGCCTTGTGAAGAA	156
GmTGA3	ACACAAGAGGCAGAAGAGGC/GACCACAGAGAACTCAGGGC	299
GmTGA4	CTAATGGTTCGCCCAGCTCA/CCTGGCAAGCCACAAAGAAC	245
GmTGA5	GGGACGGGTTCAGCATACATT/TTGACTGTGCCTGATCTCCAG	173
GmTGA6	GGATTGGTGGATTCAGGCCA/CCTCGAGTCCTTGGGAGAGA	144
GmTGA7	AAAGTTGATGGCGGTCTGAA/CCAGCTCCAATTGCATGAGC	265
GmTGA8	CGGACTCTGTAGGACCAGGA/TCCCCCAAAGCAAGCAGAAA	190
GmTGA9	TCAGCAAGTTAGCCTGTGGG/TCCAGCTCCAGTTGCATGAG	300
GmTGA10	TGGATTGGAGGATTTCGCCC/GTCCTACAGAGTCAGCGAGC	178
GmTGA11	ACACAAGAGGCAGAAGAGGC/CAGAAATGGCCACCAAGCAC	259
GmTGA12	ATGGCTCGGTGGTTTTCGAT/GAGGGCCCTAAAGAGGAGGA	198
GmTGA13	GTCCCTTGTTGCTGTGGCTT/TCTTGCAGCCTCGCGATT	100
GmTGA14	CTGAAAGGGGTGGCTGCTAA/ACGAATGCCCGAGTTCACAT	187
GmTGA15	AAAGTTTGGTTGTACTTTGCCAAC/ACACACTCATACTTCTCGGGG	284
GmTGA16	TGGACTCCGGCATTCGTTAG/ACAAGAAAACACCGTGCTGC	249
GmTGA17	GTTGCAGCAGTCACTTGTGG/GTGCTGCTTGACGAACTGTG	179
GmTGA18	TCCGGCTTTCATCTCAGCAA/GCAGCTTGAGCAGTTGTCAG	235
GmTGA19	TGCTGTCTGGCATGTGGAAA/ATGCTTCCATGCCCTGAGAC	192
GmTGA20	GTCAGGAGGGGAAAGCACTG/GCTGATCTTGGGGCATCACT	183
GmTGA21	TACGCCGTGCACTAGATGTC/GCCTTTGCAGCTTCTGCTTT	235
GmTGA22	GAAGCCCTTGCTGCTGTTTC/ATTCTGCTCTTCCAGCCACC	300
GmTGA23	GGCAGTTTGCCTGCACTGAT/TTAGAGGCATGGCTGCACTT	160
GmTGA24	GCTGCTATTGCTGGGTCTCA/CACCCCTTTCAGGCCTTTGA	175
GmTGA25	GGTTTCGTGAGACAGGCTGA/GTGCCAGCCACAAAGAACTG	148
GmTGA26	CTCTAGGGCCCTCTGGTTCT/GCTTGGCACGTGGTCAAAAT	160
GmTGA27	ATTTGCGCCAGCAGACTTTG/AGGGCACGCAACCTAGAAAA	106
GmEF1B†	CCACTGCTGAAGAAGATGATGATG/AAGGACAGAAGACTTGCCACTC	134
GmACTIN11†	ATTTTGACTGAGCGTGGTTATTCC/GCTGGTCCTGGCTGTCTCC	126

†Represent the primer sequences are originated from the literature which is reported by Ma et al. (2013) (https://doi.org/10.1371/journal.pone.0075271).

Table S3. The construction primers of transient overexpression constructs

Constructs Name	Primer sequence (5'to 3')
p103-*GFP*	Forward primer: tttggagaggacacgctcgagATGGTGAGCAAGGGCGAGG<br>Reverse primer: gactcacctaggtcacacgtgTCACTTGTACAGCTCGTCCATGC
p103-*GmTGA1*	Forward primer: tttggagaggacacgctcgagATGCCATTCTCTTCTCAAAGACACA<br>Reverse primer: gactcacctaggtcacacgtgTCACTCTCTAGGGCGCGCC
p103-*GmTGA2*	Forward primer: tttggagaggacacgctcgagATGAAAAATATGCAAGGCTTCAAG<br>Reverse primer: gactcacctaggtcacacgtgCTACTCTCTAGGGCGCGCC
p103-*GmTGA4*	Forward primer: tttggagaggacacgctcgagATGGGTAGCAGAAGTAGAACAGTAAACG<br>Reverse primer: gactcacctaggtcacacgtgTCACTCCCTTGGCCTGGC
p103-*GmTGA6*	Forward primer: tttggagaggacacgctcgagATGATGGCTTCTTCAAAGACCAC<br>Reverse primer: gactcacctaggtcacacgtgCTATTCTTGGCGAGGGCGT
p103-*GmTGA8*	Forward primer: tttggagaggacacgctcgagATGGATGCTACATCCTCACCGT<br>Reverse primer: gactcacctaggtcacacgtgCTAAGCATGTCCTGGAAATTGAGG
p103-*GmTGA10*	Forward primer: tttggagaggacacgctcgagATGGATGCTACATCCTCACAGTTTG<br>Reverse primer: gactcacctaggtcacacgtgCTAATGTTTGATGACTGACTTAGTAAGTTCT
p103-*GmTGA12*	Forward primer: tttggagaggacacgctcgagATGCAAAGCTTCAACACAACCG<br>Reverse primer: gactcacctaggtcacacgtgCTAGTACTCTCTAGGACACGCTAACCA
p103-*GmTGA13*	Forward primer: tttggagaggacacgctcgagATGGCTGACGCCAGTCCTAG<br>Reverse primer: gactcacctaggtcacacgtgTCAGTCTCTTGGGCGGGC
p103-*GmTGA14*	Forward primer: tttggagaggacacgctcgagATGGCGAGCCAAAGAATAGGA<br>Reverse primer: gactcacctaggtcacacgtgTCAGAAACTTGAGAAATGATTCTGAGA
p103-*GmTGA15*	Forward primer: tttggagaggacacgctcgagATGAATTCAGCATCCCCGC<br>Reverse primer: gactcacctaggtcacacgtgCTAAGCAGGTTCCCGGGG
p103-*GmTGA17*	Forward primer: tttggagaggacacgctcgagATGGCGAGCCACAGAATAGG<br>Reverse primer: gactcacctaggtcacacgtgTCAGAAACTTGAGAAATAATTCTGAGAA
p103-*GmTGA19*	Forward primer: tttggagaggacacgctcgagATGGGGAGTAGAACTACCTGGAGG<br>Reverse primer: gactcacctaggtcacacgtgTCAATCTCTAGGGCGAGCAAGC
p103-*GmTGA20*	Forward primer: tttggagaggacacgctcgagATGGCGAGCCACAGAATAGG<br>Reverse primer: gactcacctaggtcacacgtgTCAGAAACTTGAGAAATAATTCTGAGAA
p103-*GmTGA22*	Forward primer: tttggagaggacacgctcgagATGCCGAGCTCCAATTCTGA<br>Reverse primer: gactcacctaggtcacacgtgTCAATCTCTAGGGCGAGCAAGC
p103-*GmTGA23*	Forward primer: tttggagaggacacgctcgagATGAATTCAGCATCCCCGC<br>Reverse primer: gactcacctaggtcacacgtgCTAAGCAGGTTCACGAGGTCTATTAG
p103-*GmTGA24*	Forward primer: tttggagaggacacgctcgagATGGGTAGCAGAAGTAGAACGGTAA<br>Reverse primer: gactcacctaggtcacacgtgTCACTCCCTTGGCCTAGCAA
p103-*GmTGA25*	Forward primer: tttggagaggacacgctcgagATGGCTTCTTCAAAGACCACCA<br>Reverse primer: gactcacctaggtcacacgtgCTATTCTTGGCGAGGGCGT
p103-*GmTGA26*	Forward primer: tttggagaggacacgctcgagATGCAAAGCTTCAACACAACAACC<br>Reverse primer: gactcacctaggtcacacgtgCTAGTACTCTCTGGGACACGCTAACC
p103-*GmTGA27*	Forward primer: tttggagaggacacgctcgagATGCCGAGCTTTGATTCACAA<br>Reverse primer: gactcacctaggtcacacgtgTCAGTCTCTTGGGCGGGC
p103-*CP*	Forward primer: tttggagaggacacgctcgagATGTCAGGCAAGGAGAAAGAAGG<br>Reverse primer: gactcacctaggtcacacgtgTCACTGCTGTGGACCTATGCC
